# Aminoglycosides: Molecular Insights on the Recognition of RNA and Aminoglycoside Mimics

**DOI:** 10.4137/pmc.s2381

**Published:** 2009-04-28

**Authors:** Maruthi Chittapragada, Sarah Roberts, Young Wan Ham

**Affiliations:** Department of Chemistry and Biochemistry, Brigham Young University, Provo, UT 84602, U.S.A

**Keywords:** aminoglycosides, aminoglycoside mimics, RNA recognition, 2-deoxystreptamine (2-DOS) mimics, aminoglycoside resistance, aminoglycoside toxicity

## Abstract

RNA is increasingly recognized for its significant functions in biological systems and has recently become an important molecular target for therapeutics development. Aminoglycosides, a large class of clinically significant antibiotics, exert their biological functions by binding to prokaryotic ribosomal RNA (rRNA) and interfering with protein translation, resulting in bacterial cell death. They are also known to bind to viral mRNAs such as HIV-1 RRE and TAR. Consequently, aminoglycosides are accepted as the single most important model in understanding the principles that govern small molecule-RNA recognition, which is essential for the development of novel antibacterial, antiviral or even anti-oncogenic agents. This review outlines the chemical structures and mechanisms of molecular recognition and antibacterial activity of aminoglycosides and various aminoglycoside mimics that have recently been devised to improve biological efficacy, binding affinity and selectivity, or to circumvent bacterial resistance.

## Introduction

RNA performs pivotal biological functions when organisms, including bacterial and viral pathogens, replicate. Developing small molecules that can interact with RNA and interrupt undesired cellular activities is a promising new path for drug design.^1^ To date, RNA remains largely unexplored as a drug target and may provide untapped opportunities to develop novel therapeutics.

Antisense and RNAi strategies have been widely recognized as a powerful tool to control RNA genes and regulate development of various organisms and cellular functions of interest.^2^–^4^ However, these strategies are rather limited in practical therapeutic application due to complex and unusual RNA folding patterns and lack of efficient cellular delivery methods for antisense nucleotides.^5^ This makes development of small RNA binding molecules imperative.

RNAs can fold into complex three-dimensional structures such as loops, bulges and pseudo-knots, and consequently, they present a unique challenge for custom design of the RNA binding molecules. Although some progress has been made in recent years in understanding how small molecules recognize RNA,^6^–^8^ there are still very few examples of small molecules that bind to natural RNA structures with high affinity and specificity. So far, there has been no general rule found that can be applied to design small molecules targeting specific RNA sequences or structures.

Since they have been shown to selectively bind to a variety of RNA targets, aminoglycosides^9^ have become a central focus in an effort to understand underlying principles of RNA recognition by small-molecules. Recent NMR and X-ray crystal structures of RNAs complexed with various aminoglycosides provide significant insights on the RNA recognition by small molecules at the molecular level.^10^–^12^ Based on this structural information, many chemical modifications have been devised to improve the binding affinity and selectivity and to increase the antibacterial efficacy of existing aminoglycosides. Some modifications were aimed at synthetic aminoglycosides that are less prone to bacterial resistance while others targeted RNAs from sources other than bacteria such as viral or oncogenic RNAs. This review examines various chemical approaches taken to realize these goals.

## Chemical Structures of Aminoglycosides

**Figure 1.**
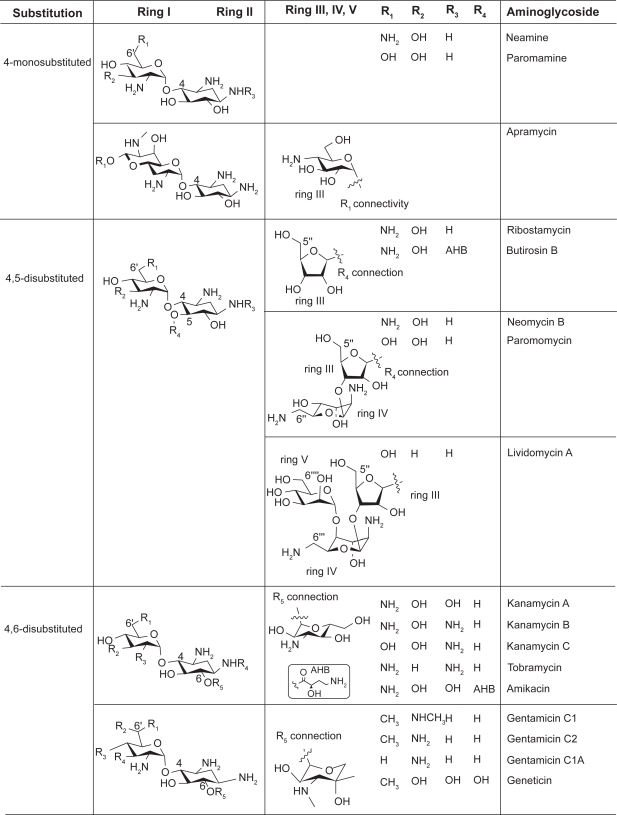
Chemical structures of the three representative classes of aminoglycosides and their substitution sites. The central scaffold 2-deoxystreptamine (2-DOS) is ring II.

Clinically significant aminoglycosides include both naturally occurring drugs and some semi-synthetic derivatives, all of which have a highly-conserved aminocyclitol ring (ring II, Figure 1), a central scaffold that is linked to various aminosugar moieties.^13^,^14^ The aminocyclitol ring is comprised primarily of 2-deoxystreptamine (2-DOS) and has 1,3-diamino functionality and three or four hydroxyl groups that provide anchoring points for aminosugars. Aminoglycosides can be divided into 3 subclasses depending on the substitution pattern: 4-monosubsituted, or 4,5- or 4,6-disubsituted (Fig. 1). Aminoglycosides in each subclass show close structural resemblance. Although 2-deoxystreptamine or 2-deoxy-*myo*-inosa-1,3-diamine is actually derived from D-glucose-6-phosphate biosynthetically, the numbering system is based on streptamine’s biogenic precursor *myo-*inositol as described by Fletcher.^15^

Neamine, paromamine, and apramycin fall under the 4-monosubstituted aminoglycosides. Paromamine and neamine differ only in the R_1_ substituent and are typically not used alone as drugs, leaving apramycin being the only 4-monosubstituted compound that is actually used pharmaceutically. Apramycin is unique in that its ring I is bicyclic.

Ribostamycin, butirosin B, neomycin B, paromomycin, and lividomycin A belong to 4,5-disubstituted aminoglycosides. These compounds can be further divided into three groups based on the additional ring III and its substituents. Lividomycin A is the only aminoglycoside in this category that contains a fifth ring.

4,6-disubstituted aminoglycosides includes kanamycin A, kanamycin B, kanamycin C, tobramycin, and amikacin in one category and gentamicin C1, gentamicin C2, gentamicin C1A, and geneticin in another. The major differences between these two subclasses is the variation of an extra R group attached to the 6’ position of ring I as well as the ring III connected to the 2-DOS.

## Recognition of RNAs by Aminoglycosides

Aminoglycosides have been recognized to have binding capabilities with many different functional RNAs such as the prokaryotic ribosomal A-site,^16^–^18^ HIV TAR,^19^ HIV RRE,^20^ Group I intron,^21^ RNase P,^22^ tmRNA^23^ and the eukaryotic A site^24^ even in some cases with relatively low micromolar binding affinities.

The binding of the aminoglycosides to these target RNAs is mediated through two different interactions: (1) hydrogen bonding between amino and hydroxyl functional groups of aminoglycosides and RNA bases,^25^ and (2) electrostatic interactions between the negatively charged phosphate backbone of the RNA and positively charged amino functional groups of the aminoglycosides.^26^ The latter dominates the interactions between aminoglycosides and target RNAs due to the presence of multiple amines among the aminosugar rings (ring I, III, IV and V), which makes RNA-aminoglycoside binding highly promiscuous. For instance, neomycin binds to bulge regions of unrelated RNA sequences from the 16S ribosome,^27^ HIV TAR,^28^ HIV RRE,^20^,^29^ and Group I intron^30^,^31^ with affinities in the low micromolar range. The ambiguous binding characteristic of aminoglycosides originates not only from the electrostatically driven binding mode but also from inherent conformational adaptability of aminoglycosides.^32^ The glycosidic connection assumes a variety of conformations and permits aminoglycosides to structurally adjust to diverse RNA targets.

Experiments have shown that aminoglycoside binding is somewhat dependent on the size of an asymmetric interior loop of the target RNAs rather than its sequence.^33^ These examples demonstrate that the design of small RNA-binding molecules is highly dependent on experimental trials and makes sequence- and/or site-specific recognition by small molecules a very difficult task.

## Highly Conserved Interactions Observed by the Ring I and Ring II (2-DOS)

**Figure 2.**
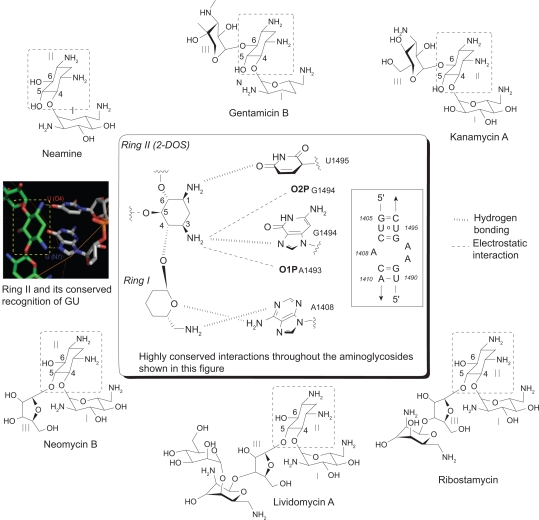
Sequence-specific recognition of A-site RNA by rings I and II (2-DOS) of six aminoglycosides of different sizes and substitution patterns. Highly conserved hydrogen-bonding (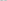) and electrostatic interactions (----) are indicated with dashed lines.

Despite the convincing facts that aminoglycosides recognize RNAs in a promiscuous manner, studies suggest that there are some sequence-dependent elements in the recognition of target RNAs. A wide variety of RNAs, such as A-site rRNA, ribozymes, and HIV-1 RRE and TAR, are known to bind to various aminoglycosides. These RNAs contain 5′-GU-3′ or 5′-GG-3′ base steps in common next to the nucleotides in bulges or in noncanonical stem structures. A similar binding pattern was observed when A-site RNA sequence was treated with aminoglycosides of various sizes and substitution patterns on ring II. Specifically, the crystal structures of six aminoglycoside antibiotics (neamine, gentamicin C1A, kanamycin A, ribostamycin, lividomycin A and neomycin B) and oligonucleotides containing the decoding A site of bacterial ribosome revealed that rings I and II (2-DOS) are essential for recognition of A-site RNA, and interactions between the rings I and II of all aminoglycosides and the target RNA are highly conserved and sequence-specific (Fig. 2).^11^ The two amino groups of the ring II (2-DOS) unanimously recognizes the 5′-GU-3′ base step (bold) in A-site through hydrogen bonding with N7 of G1494 and O4 of U1495 and electrostatic interactions with negatively-charged phosphate backbone that are conserved throughout all six aminoglycosides. The puckered sugar ring I is inserted into the A site helix by stacking against G1491 and forms a pseudo base pair with two hydrogen bonds to the Watson-Crick sites of the universally conserved A1408. This clearly suggests that contacts made by the ring I and ring II (2-DOS) of various aminoglycosides to the target RNA are somewhat independent of their structural context of aminosugar subunits and more dependent on helical sequence.

The sequence-specific recognition pattern of 2-DOS is further supported by the following mutation study. A mutation of G1494A in the target site was deleterious for paromomycin binding because it prevented specific hydrogen bonding of 2-DOS to the RNA.^18^ This result indicates that the 2-DOS ring of paromomycin strictly recognizes the 5′-G(N7)-U(O4)-3′ sequence but not 5′-A(N7)-U(O4)-3′, probably because of a steric clash between the bulky amino group of A and the 2-DOS moiety.

**Figure 3.**
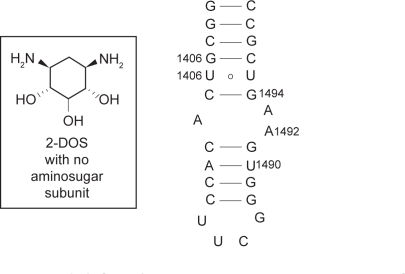
Recognition of 5′-GU-3′ base sequence step by the 2-DOS monomer that does not have any aminosugar substituent.

Strikingly, the same binding pattern of 2-DOS was demonstrated when Puglisi and coworkers treated isolated 2-DOS, without the aminosugar subunit attached, with wild type A-site of 16S rRNA (Fig. 3).^12^ The binding activity between 2-DOS and A-site RNA was monitored by high-resolution NMR techniques. The 2-DOS specifically recognized the two 5′-GU-3′ base steps (G1494-U1495 and G1405-U1406) even though their binding affinity is low (∼1 mM). A titration experiment with the RNA whose A1492 bulge nucleotide was deleted confirmed that the bulge residue of A-site RNA was required to open the major groove for accommodation of the deoxystreptamine molecule.

## 2-DOS Mimics

**Figure 4.**
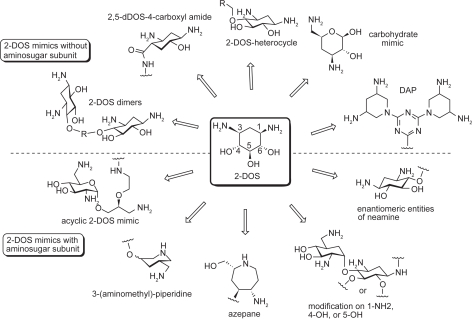
The 2-DOS mimics without or with an aminosugar subunit. The 2-DOS mimics with an aminosugar subunit use mostly neamine as the structural platform for modification.

Universal existence of the 2-DOS moiety at the central position of almost all clinically important aminoglycosides suggests its crucial role in RNA recognition and biological activity. Therefore, mimicking the 2-DOS structure has been of great interest in preparing novel aminoglycoside analogs. The 2-DOS mimics that have been reported so far can be divided into two different groups based on whether an aminosugar subunit was included in the mimic or not (Fig. 4).

Although most 2-DOS mimics are based on the neamine backbone as the building platform, some use 2-DOS as the sole component, as 2-DOS alone has demonstrated RNA-binding capability without the assistance of aminosugars. Wang et al. demonstrated this example (**1**) by removing aminosugar rings from the original aminoglycoside structures, leaving only the core 2-DOS as the main constituent of the compound.^34^ This compound demonstrated strong affinity (*K_d_* value of 88 μM) for the target RNA A-site and inhibited bacterial translation. This example supports the idea of generating small synthetic compounds that mimic the conformation and function of aminoglycosides without using aminosugar residues.

Following are examples that use 2-DOS as the central scaffold and replace the aminosugar ring I of neamine with other structural motifs. Ding and coworker prepared heterocyclic 2-DOS derivatives 2 based on modeling studies.^35^ Most 2-DOS analogs demonstrated a modest increase in binding affinity upon conjugation with a variety of heterocycles, while the binding affinity of the compound containing an electron-withdrawing group (CF_3_) did not show any increase.

Hermann et al prepared a small library of monomeric 2-DOS analogs **3** conjugated with non-sugar heterocycles through an amide bond (Fig. 7).^36^ Although some compounds show slightly improved binding affinity to the target RNA A-site of bacterial 16S RNA, most did not, suggesting that the rigid amide linkage locked the non-sugar scaffold in an unfavorable conformation for the recognition of the target RNA.

3,5-Diamino-piperidinyl triazines (DAPT) **4** were reported as novel antibacterial translational inhibitors using *cis*-3,5-diaminopyridine (DAP) to mimic the 2-DOS scaffold.^37^ Many of the DAPT compounds that behaved as potent inhibitors of bacterial protein synthesis did not show corresponding antibacterial activity. The potency of the DAPT compounds in the antibacterial activity was finally achieved when the R_2_ substituent was replaced by aromatic structures that facilitated better cell membrane penetration. Recently, van Delft and coworkers reported a carbohydrate mimic of the 2-DOS that can be used to prepare conformationally constrained aminoglycosides.^38^

Aminoglycosides like neomycin show binding affinity to various RNA targets such as the 16S ribosome,^39^ HIV TAR,^40^ and HIV RRE,^20^ but do not typically bind to RNA hairpin loops, which are a major RNA secondary structural motif. However, when Hergenrother and coworkers prepared simple dimers of 2-DOS (**5**) by connecting two 2-DOS molecules with various aliphatic and aromatic linkers of different sizes, they were found to bind tightly to RNA hairpin loops of various sizes from 4 to 8 nucleotides, most of which contain 5′-GU-3′ sequence.^41^ The dimers with the aromatic linkers exhibited slightly tighter binding over their aliphatic counterparts.

These examples show that novel RNA binding molecules may be built solely based on 2-DOS without the assistance of conjugated aminosugar(s), and the intrinsic RNA binding affinity of the 2-DOS may be useful targeting not only helical RNAs whose base pairing is disrupted, but also RNAs of other types of secondary structure such as loops that conventional aminoglycosides do not bind.

Many other 2-DOS mimics were prepared based on the neamine backbone. To have a better understanding of the effect of stereospecificity of aminoglycosides, Rando and co-workers synthesized and compared the activity of (+)-neamine **6**, (−)-neamine ***ent*-6**, its 5-positional isomers **7**, ***ent*-7** as well as 6-positional isomers **8**, ***ent*-8**.^42^ The enantiomeric series ***ent*-8** was found to be the most effective inhibitor among this series, which allows 1-NH_2_ and 3-NH_2_ to face the active site. The isomer ***ent*-6** with both amino groups pointing away from the active site exhibited the least potent activity, while **7** and ***ent*-7** with only one of the amino groups away from active site exhibited less binding affinity.

In an effort to mimic the unique spatial arrangement of the two amino functional groups in 2-DOS, the Hermann group prepared two series of compounds by replacing the 2-DOS in neamine with azepane- and piperidine-based structures. Azepane-glycosides **9** and **10** exhibited moderate antibacterial activity against Gram-positive *Staphylococcus aureus* and retained this activity against aminoglycoside-resistant strains.^43^ This result substantiates the importance of the bacterial decoding site as a valuable target for future development of novel antibiotics. Compounds **11** and **12**, designed based on 3-(aminomethyl)-piperidine,^44^,^45^ demonstrated higher activity and specificity towards the bacterial targets than eukaryotic RNAs.

**Figure:**
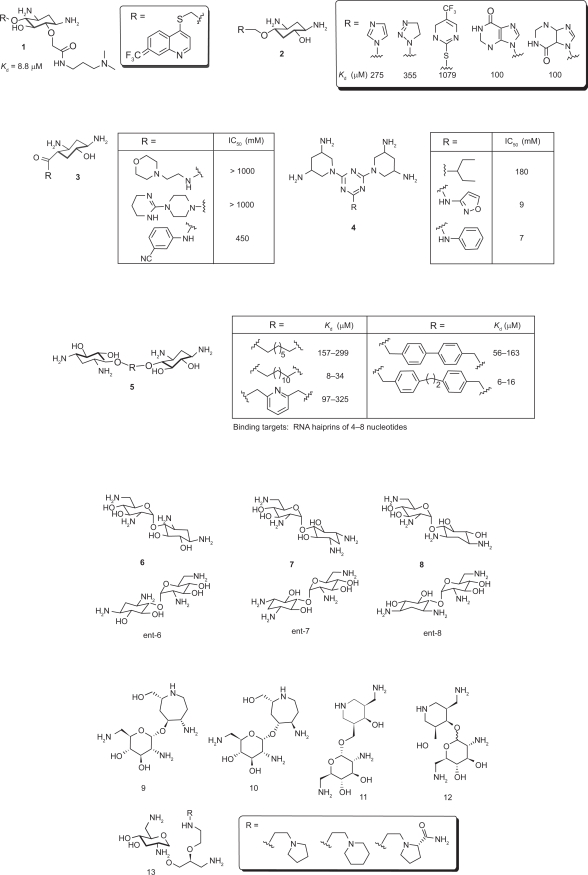


**Figure:**
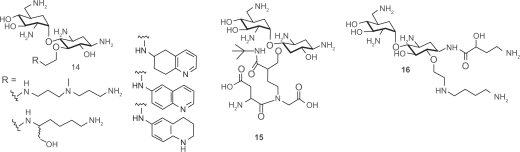


While conformationally-restricted azepane- and 3-(aminomethyl)-piperidine-based compounds **9**–**12** showed moderate antibacterial activities, the acyclic 2-DOS mimics **13** that have increased flexibility have a detrimental effect on their RNA binding efficacy.^46^

Since Wong and coworkers found that the 5-position is sensitive for aminoglycoside-RNA recognition, small libraries of neamine mimetics were reported with various substituents at the 5′-OH position. Polyamine and 6-aminoquinoline analogs **14** demonstrated a high affinity for the oncogenic Bcr-Abl and PAX3-FKHR single-stranded mRNA breakpoint and a capability to regulate gene expression.^47^ This provides evidence that aminoglycosides can be used to target not only bacterial and viral but also oncogenic mRNAs. Short semi-peptidic moieties were also introduced on the 5′-OH position of the neamine scaffold by combinatorial synthesis to form analogs like **15** in an attempt to inhibit interactions between HIV-1 RRE and Rev protein. But, the inhibition activity was only slightly better than neomycin B.^48^ Other neamine analogs including **16** were reported with modifications at 6′-OH and 1-NH_2_ positions.^49^ The substituent on 1-NH_2_ was adopted from another aminoglycoside butirosin. This compound **16** was found to be especially interesting because it appears to be uncompromised by aminoglycoside-resistant enzymes and show a considerable increase in antibacterial activity. The crystal structure of the complex between **16** and A-site of the ribosome suggested that existence the AHB substituent on the 1-NH_2_ hinders proper complex formation with key aminoglycoside-modifying enzymes.

## Aminosugar and Aminoglycoside Mimics

**Figure 5.**
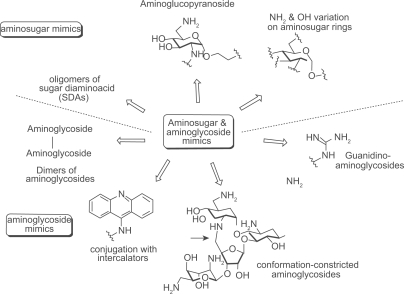
Aminosugar and aminoglycoside modifications.

A number of synthetic aminosugars and aminoglycosides have been prepared in an effort to accomplish several goals including: mimicking the core aminosugar rings for enhanced antibacterial activity, limiting the overall structural flexibility of the aminoglycosides, and addressing recognition of the conventional aminoglycosides by the aminoglycoside-modifying enzymes in resistant strains (Fig. 5). Below are relevant examples of aminosugar and aminoglycoside modifications.

One of the aminosugar mimics was achieved by −NH_2_ and −OH functional groups at various positions of ring I, II and IV of aminoglycosides to find optimal substitution patterns for aminosugars. Wong and coworkers prepared a series of compounds by placing −NH_2_ and −OH functional groups at various positions of the ring I in neamine and found that 2′,6′-diamino substitutions on ring I to be the most effective A-site binder.^50^ The mono-amino derivative **17** showed activity against *E. coli, P. aeruginosa,* as well as *S. aureus*. However, its MIC was less than that of neamine, which can be explained by the loss of the highly conserved hydrogen bonds and electrostatic interactions observed in crystal structures. This suggests that neamine is the most promising pseudodisaccharide core that should be kept for further development.

Similar approaches have been performed on the ring III of tobramycin by replacing the ring with various mono- or diaminosugars.^51^ The tobramycin derivatives were tested for binding affinity with several conserved RNA sequences from bacterial and viral sources. Surprisingly, no direct correlation was observed between the dissociation constants and the number of charges on the derivatives, considering that the binding of aminoglycosides to RNA targets is driven by electrostatic interactions to a large extent. Chang and coworkers replaced the ring III of pyranmycin with various L-aminosugars and L-pyranoses.^52^

**Figure:**
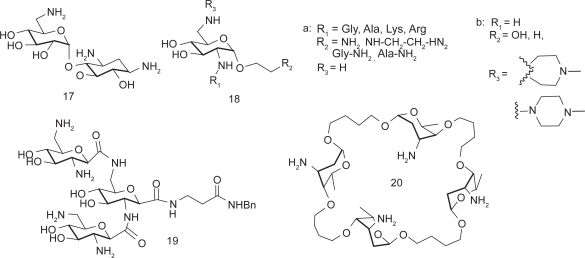


Some aminosugar mimetics did not include the 2-DOS core in its modified structures. A library of compounds such as **18** have been developed for the discovery of small molecules using a 1,3-hydroxyamine motif.^53^–^55^ Wong and coworkers prepared a library of compounds **18a** by substituting R_1_- and R_2_-positions with highly basic amino acids and amine-containing linear chains, repectively.^54^ This library of compounds recognized both wild type 16 S rRNA and several related mutant sequences, which proved that the hydroxylamine core motif is ideal for the design of high affinity RNA binders. However, when Wengel and coworkers introduced substituents on R_2_- or R_3_-positions to make compounds like **18b**, they were only minimally active against various target RNAs.^53^ Sugar diamino acids (SDAs) with unprotected amino groups represent a new class of potential aminoglycoside mimetics.^56^ The synthesis of the first examples of oligosaccharide mimetics such as **19** suggest that a high degree of diversity can be achieved by conjugating only a small set of different SDAs through amide bond.

**Figure:**
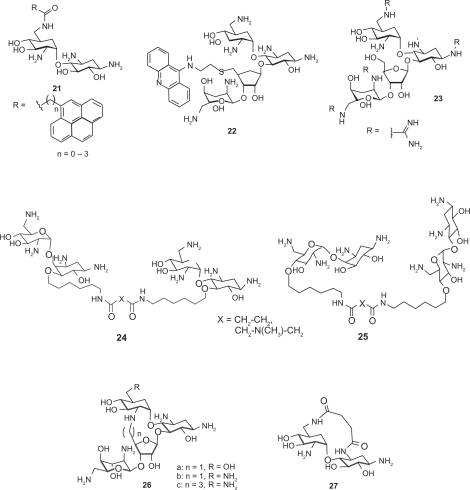


Carlomagno and coworkers reported a neooligoaminodeoxysaccharide **20** as a novel aminolgycoside analogue by connecting four aminosugar rings using linear alkyl linker.^57^ Unlike other aminoglycosides, this compound was able to make simultaneous contacts with the bulge residues required for Tat binding and A35 residue of the hexanucleotide loop of HIV-2 TAR RNA.

Most aminoglycoside mimics have been prepared by adding extra functionality onto the original aminoglycoside structures without removing aminosugar substituents. A number of intercalating agents were included in the extra functionality with the anticipation that aromatic compounds may have an intercalating capability to the RNA targets that aminoglycosides typically bind. Hamasaki and coworkers conjugated acridine, anthracene and other similar aromatic structures to the ring I of neamine.^58^ Pyrene-conjugated neamine analogs **21** demonstrated up to 87 times stronger binding affinity to HIV RRE mRNA than neamine. Neomycin B was also conjugated with acridine at its 5″-position on the ring III via a short linker. Conjugate **22** was one of the strongest competitive inhibitors of HIV rev-RRE interaction, which came at the expense of losing binding selectivity to RRE.^59^ Quinacridine conjugated with two aminoglycosides, used as a potential telomerase activity modulator targeting the P6.1 element of RNA telomerase, was another example of intercalator-conjugates.^60^

Guadinylation of multiple amino groups of aminoglycosides shows similar effects as intercalating agents. Guanidylated neomycin B (**23**) was reported to inhibit the viral replication 100 times more potently than neomycin B by targeting HIV RRE.^61^,^62^ A similar result was also observed with tobramycin.^60^ It is notable that aminoglycosides generally exhibit poor uptake by eukaryotic cell lines but guadinylation of the aminoglycosides dramatically enhanced cellular uptake of aminoglycosides.^63^ Lapidot and coworkers reported high inhibition activity against HIV-1 by conjugating arginine to introduce the guanidine functionality to neomycin B.^64^

Multivalent aminoglycosides have been reported to target RNA structures such as HIV-1 TAR RNA due to their unique structure that allows for multiple binding interactions. Four symmetrical 4′,4′- and 5,5-neamine dimers **24**–**25** were prepared and studied for their affinity toward TAR RNA using fluorimetric binding assays.^65^ All these dimers inhibited TAR-Tat binding at submicromolar concentrations. Trimeric neamine was also prepared but not useful for activity studies as it resulted in precipitation of the TAR RNA. Other reported dimeric aminoglycosides^66^ (neomycin-neomycin, neomycin-tobramycin, and neomycin-kanamycin A) demonstrated much higher inhibitory activity than exhibited by doubling the concentration of each aminoglycoside due to the favorable entropic factors.

The lack of high RNA target selectivity of aminoglycosides is partially attributed to their conformational flexibility that allow adjusting to different shapes of RNAs. Tor and coworkers prepared conformationally-constrained neomycin and paromomycin derivative **26a** and **26b** by covalently connecting two aminosugars (ring I and ring III) to reduce the number of available conformations, thus changing target selectivity.^32^ However, the conformational constraint did not help in discriminating different RNA targets, which suggests that the structure of the target RNA and its flexibility also play an important role in the binding event. Mobashery and coworkers reported another kind of structurally constrained aminoglycoside **27** based on neamine.^67^ However, no biological activity was reported for this compound.

Arya and coworkers reported an oligonucleotide-neomycin conjugate for sequence-specific targeting of RNA. The nucleotide-neomycin conjugate demonstrated enhanced duplex formation for its target RNA α-sarcin loop.^68^

While most aminoglycoside mimetics employed single point modification on one of the rings of aminoglycosides, Houston and coworkers reported multisite modification on the structure of neomycin B.^69^ Three rings (rings II, III, and IV) of the neomycin B were modified at the same time using Mitsunobu and Click chemistry.

## Aminoglycoside Resistance

The increasing bacterial resistance to clinically important aminoglycosides catalyzed the search for novel aminoglycoside mimics. Among several different resistance mechanisms, bacterial inactivation of aminoglycosides by intracellular aminoglycoside-modifying enzymes is the most significant source of resistance development.^70^ More than 50 different types of aminoglycoside-modifying enzymes have been found in resistant bacteria. These enzymes modify aminoglycosides through acetylation of amino groups (N-acetyltransferases, AAC), and adenylation (O-adenyltransferase, ANT) and phosphorylation (phosphotransferases, APH) of hydroxyl groups on aminoglycosides.

According to recent studies mainly focused on tobramycin, kanamycin A and B, amikacin, gentamicin, and geneticin, differences in ring III did not seem to alter the interaction of the drug with target rRNA, but subtle variations of ring I significantly influenced binding. Ring II is also affected by the enzymes. The 2′-and 6′-NH_2_ groups of the ring I and 3-NH_2_ of the ring II are prone targets for acetylation.^16^ These three amino groups make highly conserved interactions with A-site RNA. The two hydroxyl groups at 2′- and 4′-positions of ring I are targets for adenylation and phosphorylation. When these important functional groups are recognized by the enzymes and modified by one of the three reactions, their highly conserved interactions, both hydrogen bonding and electrostatic interactions, are prevented and ultimately cause aminoglycosides not to be able to bind to RNA targets.

**Figure 6.**
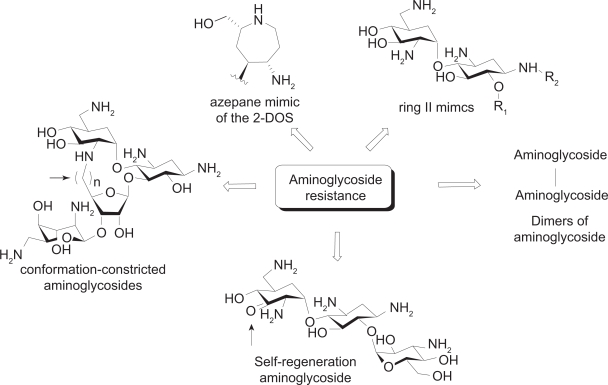
The aminoglycoside mimics that are designed or found to be effective against resistant bacterial strains.

One way to meet the challenge presented by aminoglycoside resistance is to discover new aminoglycoside analogs that are less prone to enzymatic modification. Following are some examples of the aminoglycoside mimics that are designed rationally to fight against aminoglycoside resistance or that are found to be effective against resistant bacterial strains (Fig. 6), many of which are already discussed above.

Various azepane mimics of the 2-DOS including **9** and **10** demonstrated moderate antibacterial activity against resistant strains as well as *S. aureus.*^43^ This experiment indicates that the biological activity of the natural antibiotics can be maintained while the structural difference of the designed scaffold from the natural antibiotics help escape some resistance mechanisms.

The ring II mimic of neamine (**16**) with substitutions at 1-NH_2_ and 6-OH revealed potential structural aspect that may be used to target resistant strains.^50^ Its crystal structure suggests that the aminohydroxybutyryl (AHB) substituent on the 1-NH_2_ position may cause steric clash with the aminoglycoside-binding loop of the aminoglycoside phosphotransferase APH(3′)-IIIa and prohibits proper complex formation.

Dimers of neamine and nebramine were reported to be active against several aminoglycoside resistant bacterial strains, especially for the treatment of *P. aeruginosa* infection.^71^,^72^ Interestingly, the length of the linker of the dimers was found to be an important determinant of antibacterial activity of the bivalent aminoglycosides.^72^

Asensio and coworkers also prepared conformationally-restricted derivatives of neomycin **26a** and **26c** and examined whether they would be susceptible to enzyme inactivation.^73^ The modification provided an effective protection against aminoglycoside inactivation enzymes involved in aminoglycoside resistance, which represents a test case that a structure-based approach may be used to design ligands that maintain binding affinity to desired RNA targets but a poor substrate for enzymes that lead to inactivation.

Distinct from all of the above examples that are designed to avoid interactions with resistance enzymes, Mobashery and coworkers designed an aminoglycoside that binds to the resistance enzyme APH(3′) but not affected by its enzymatic activity.^74^ The versatile aminoglycoside kanamycin A was made clinically obsolete by the widespread expression of resistance enzymes APH(3′)s in pathogens. These enzymes phosphorylate the 3′-OH of amino-glycosides. In the design, the 3′-OH was oxidized to ketone **28**, which in turn goes through (1) hydration to give geminaldiol, (2) phosphorylation of the hydroxy group by APH(3′), then (3) self regeneration of the ketone by dephosphorylation. While the MIC of kanamycin A increased 500 to 1000 fold when treated with resistant *E. coli*, the self-regenerating analog **28** has shown only 4-fold increase of MIC toward resistant *E. coli*, which signifies that the self-regenerating strategy is a feasible approach in lowering MIC for resistant strains.

**Figure:**
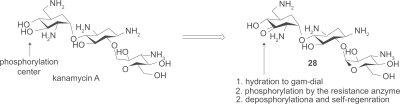


## Medicinal Aspects: Mechanisms of Antibacterial Activity and Toxicity

In addition to being a useful model in understanding the RNA recognition process by small molecules, aminoglycosides have been used as versatile antibiotics for almost six decades due to their enormous therapeutic value. For instance, gentamicins and amikacin are used to treat meningitis, pneumonia, and sepsis. Paromomycin is used for amoebic dysentery^75^ and neomycin is used for ulcers and dermatitis.^76^

Aminoglycosides exert their antibacterial activities by two different mechanisms. The first is by inhibiting the translation of essential proteins for bacterial growth. The binding of aminoglycosides to bacterial 16S rRNA, as shown in Figure 2, stops the translocation of the peptidyl-tRNA from A-site to the P-site resulting in the misreading of the mRNA. Thereby, prevention of the production of the essential bacterial proteins leads to bacterial cell death.^77^ However, this binding mechanism alone does not explain the significant bactericidal effect of aminoglycosides because other antibiotics like tetracycline that function in a similar manner are not as bactericidal. Secondly, aminoglycosides, being highly positively charged, interact with negatively charged outer membranes of Gram-negative bacterial cells through electrostatic interaction and disrupt membrane integrity by displacing Mg^2+^ and Ca^2+^ bridges that connect neighboring lipopolysaccharides.^78^ This creates temporary openings in the membrane and results in leaking of intracellular contents and increased antibiotic uptake through the membrane. This transport across the cytoplasmic membrane is known to be oxygen-dependent, thus aminoglyco-sides are less effective in anaerobes.^79^ As a result, aminoglycosides are active primarily for aerobic Gram-negative bacteria such *Pseudomonas*, *Acinetobacter,* and *Enterobacter* and some Gram-positive bacteria.

**Figure 7.**
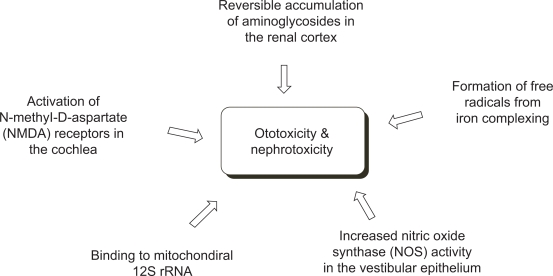
Major mechanisms of toxicity of aminoglycosides.

Despite the versatile antibacterial application against a broad-spectrum of bacteria, the high level of ototoxicity and nephrotoxicity of the aminoglycosides often limits their use in broader applications. While nephrotoxicity is known to originate from reversible accumulation of amino-glycosides in the renal cortex,^80^ the ototoxicity has shown more complex mechanisms such as binding of aminoglycosides to mitochondrial 12S rRNA,^81^,^82^ increase in nitric oxide synthase activity in the vestibular epithelium,^83^ activation of N-methyl-D-aspartate (NMDA) receptors in cochlea,^84^ and formation of free radicals from complex formation between aminoglycosides and iron^85^ (Fig. 7). In cases of the last two examples, it was demonstrated that respective use of antagonist for the NMDA receptor^86^ and free radical scavengers^87^ or iron chelators^85^ alleviated the toxicity.

Several toxicity elements of aminoglycosides were identified in relation to structures of aminoglycosides. These were determined based on effect of the number of amino groups available in aminoglycosides and compared to the toxicity of clinically used aminoglycosides and their derivatives, Baasov and coworkers pointed out that decreases in the number of amino groups in a given aminoglycoside resulted in lower toxicity.^88^ In addition to the number of amino groups, the basicity of a given amino group was an important factor in toxicity. Introducing an electron-withdrawing fluoro group in place of 5-OH of the 2-DOS ring of amikacin and other aminoglycosides effectively decreased toxicity as it decreased the basicity of the neighboring amino groups.^89^ A similar result was observed with the 2′-NH_2_ group located on the ring I of neamine, gentamine, and other aminoglycoside analogs.^90^ Removal of the 3′-OH of kanamycin B, which is located next to the 2′-NH_2_, increased the toxicity dramatically, while removal of the relative distant 4′-OH had a limited effect on toxicity. This is also understood by the basicity change of the neighboring amino group. Owada reported that N-acetylated amino-glycosides possess considerably lower toxicity than their free aminocompounds.^91^

Recently, a significant discovery was reported regarding the toxicity of gentamicin. Sandoval and coworkers isolated each of four congeners (C1, C1a, C2, and C2a) of gentamicin using HPLC from a native gentamicin sample and found that gentamicin C2 does not have nephrotoxicity but retains the native antimicrobial efficacy.^92^ It is notable that different substitution pattern on only one ring (ring I) can make the difference in the toxicity profile.

## Conclusion

The unique and complex three-dimensional structures that RNA forms provide unmet opportunities to design novel therapeutics targeting various RNAs. However, it has been generally believed that the intrinsic flexibility of RNA structures makes the structure-based rational drug design approach, which resulted tremendous success in protein-targeting therapeutic development, less suitable unless the target RNA is locked into a rigid conformation, which is not the case for most mRNAs. Despite the unfavorable view, the examples described in this review demonstrate that the structure-based drug design approach has been instrumental to a great degree in understanding the fundamental principles that govern the interactions between various RNA targets and small organic molecules and to design aminoglycoside mimics with better target selectivity and/or binding affinity. Thus, structure-based drug design will continue to play a significant role in meeting the challenges to overcome bacterial resistance to natural aminoglycoside antibiotics and design novel RNA-binding ligand targeting RNAs other than bacterial sources. The highly conserved sequence-specific recognition pattern of rings I and II of neamine, a common component for many clinically important aminoglycosides, may be put to use through the rational drug design approach to help devise small molecules that recognize specific sequences or sites of RNA. High throughput screening approaches, combined with the rational design, may offer fresh insight into the design of novel ligands, especially in identifying target-specific ligands.

In addition to aminoglycosides, riboswitches may serve as another important model in understanding the principles that govern small molecule-RNA recognition. Riboswitches are embedded within 5’-untranslated regions (5’-UTR) of certain bacterial mRNAs and regulate gene expression in response to small cellular metabolites. Since their discovery in 2002,^93^ more than a dozen classes of riboswitches have been discovered. Recent work suggests that certain antibacterial compounds discovered decades ago exert their antibacterial activity by targeting riboswitches,^94^–^96^ which establishes the viability of this approach for novel antibiotics. The fact that riboswitches form structurally complex and highly selective receptors for a given metabolite should allow highly active compounds to be designed that target riboswitches without interfering with other cellular RNAs or proteins,^97^ which may provide wealth of information in RNA targeting using small molecules.
